# Dissociative Electron Attachment From Vibrationally Excited Molecules in Nanosecond Repetitively Pulsed CO Discharges and Afterglows

**DOI:** 10.3389/fchem.2019.00163

**Published:** 2019-03-29

**Authors:** Lucia Daniela Pietanza, Gianpiero Colonna, Mario Capitelli

**Affiliations:** P.Las.M.I. Lab, CNR-Nanotec, Bari, Italy

**Keywords:** nanosecond pulsed discharges, afterglows, CO vibrational distribution, electron energy distribution function, dissociative electron attachment, global rates

## Abstract

Non-equilibrium vibrational distributions and electron energy distributions of CO in nanosecond repetitively pulsed (NRP) discharges and afterglows have been determined from a coupled solution of the time dependent Boltzmann equation for the electron energy distribution function (eedf) of free electrons and the master equations for the vibrational distribution function (vdf) of CO and the electronic excited states of CO and O and C atoms. Emphasis is given to the role of dissociative electron attachment (DEA) from vibrationally excited states in affecting the eedf and vdf under extreme conditions, i.e., an optically thick plasma with quenching processes involving the electronic excited states, populated by a sequence of discharge pulses and corresponding afterglows. In particular, the quenching process of the a^3^Π electronic state of CO determines a pumping of vibrational quanta in the ground state, which in turn largely modifies the CO vdf promoting the activation of DEA process. DEA rate coefficients have been obtained by using a complete set of vibrational (v) dependent cross sections through the *CO*^−^(*X*^2^Π) channel and by using the experimental *v* = 0 cross section of Rapp and Briglia, which should include the contribution of other *CO*^−^ resonant states. The importance of the last contribution has been also estimated by using a scaling law to extend the *v* = 0 cross section over all the vibrational ladder of CO. In particular, this mechanism becomes competitive with the other reactive channels for very short inter-pulse delay times, i.e., the *t*_*id*_ = 1 μ*s*, being less important for longer inter-pulse delay times, i.e., the *t*_*id*_ = 25 μ*s*.

## Introduction

Non-equilibrium plasma kinetics is a topic of large interest for many applications in different fields such as plasma chemistry, plasma and laser physics, hypersonic and shock wave flows (Capitelli et al., 2016). Particular attention is paid to the development of kinetic models which couple the Boltzmann equation for the electron energy distribution function (eedf) with the state-to-state vibrational kinetics for the calculation of the vibrational distribution function (vdf) of molecules and the collisional-radiative models for the electronic excited state densities. This approach become essential when the chemistry at the basis of the relevant application is dependent on the high lying vibrational levels of the considered molecules (Capitelli et al., 2016).

An example in this direction is represented by the formation of negative H^−^ ions in magnetic multi-cusp H_2_ plasmas (Bretagne et al., 1985; Hassouni et al., 1998; Capitelli et al., 2006) and more in general in RF discharges to be used as negative ion beam source for neutral heating in tokamak devices. In this case, the dissociative attachment from highly excited vibrational levels rather than from the ground state vibrational level is responsible of negative ion production.

A second example is represented by expanding hypersonic and shock wave flows where the correct description of the dissociation process depends on the whole vibrational distributions of considered molecules (Capitelli et al., 2016).

A third example, largely investigated in the present days, is the activation of CO_2_ in cold plasmas, where the dissociation process involves either the electron impact dissociation process of asymmetric mode of CO_2_ or the heavy particle dissociation processes assisted by vibrational excitation (Capitelli et al., 2017). In addition, the reacting CO_2_ plasma forms CO and O_2_ molecules that in turn undergo a complicated non-equilibrium vibrational kinetics coupled to the Boltzmann equation for eedf. The description of CO_2_, CO, O_2_ vdf's needs of accurate sets of state-to-state cross sections which requires the intensive use of quantum chemistry and molecular dynamics methodologies (Capitelli et al., 2016; Barreto et al., 2017). This last aspect has been in particular developed by Laporta et al. (2012, 2014, 2016) which have calculated complete sets of electron molecule cross sections including dissociative attachment from the whole vibrational ladder of CO and O_2_. The set of O_2_ cross sections have been recently used by Annusova et al. (2018) for O_2_ discharges operating at low pressure.

In this contest, nano-repetitively pulsed (NRP) CO discharges, fed by a sequence of modulated ns pulses followed by the corresponding afterglow of different durations, have been recently investigated (Pietanza et al., 2018a,b). To this end, a self-consistent model based on the coupling of the Boltzmann equation for the electron energy distribution function (eedf), the vibrational kinetics and the plasma chemistry of reacting mixture has been used (Capitelli et al., 2016; Pietanza et al., 2017a,b, 2018a,b).

Four models were considered in Pietanza et al. (2018b) depending on the hypotheses on the processes involving the electronic states of CO and of oxygen and carbon atoms. In particular, we have considered: (1) an optically thick CO plasma, with and without quenching processes and (2) an optically thin CO plasma, with and without quenching processes. The thick case assumes that all the spontaneous lines emitted by the CO, O and C electronic excited states are completely re-absorbed, while in the thin case such radiation totally escapes from the plasma.

Among the quenching processes included in the model, particular emphasis was given to the quenching process involving the metastable *a*^3^Π state of CO, which was also supposed to pump the vibrational *v* = 27 level of the ground electronic state of CO (Pietanza et al., 2017a,b, 2018a,b).

The previous different models predict different time dependent behavior of the electronic excited state population with a direct consequence on the eedf and on the electron impact rate coefficients and an indirect one on the vdf.

In the previous papers (Pietanza et al., 2018a,b), we neglected the role of dissociative electron attachment (DEA) of CO in the kinetics. This assumption is justified for conditions where the reacting mixture does not contain appreciable concentrations of high lying vibrational levels. The DEA process through the *X*^2^Π resonant channel of *CO*^−^, labeled as DEA(*X*^2^Π), i.e., the process

(1) $$\begin{array}{c} e+CO\left(X^{1}\Sigma^{+},v\right)\to CO^{-}\left(X^{2}\Pi \right)\to C{(}^{3}P)+O^{-}{(}^{2}P) \end{array}$$

presents a small *v* = 0 cross section, which, however, exponentially increases with the increase of vibrational quantum number *v*, as recently shown by Laporta et al. (2016).

The results of Laporta et al. (2016) do not include the DEA process through the other resonant states of *CO*^−^, in particular the *A*^2^Σ state. The experimental *v* = 0 cross section measured by Rapp and Briglia (1965) and reported by Itikawa (2015), which should include such contributions, is much higher than the *v* = 0 cross section involving the state *X*^2^Π considered by Laporta et al. (2016). No data are at the moment available for the dependence of the experimental cross section on v, which however should be weaker than the corresponding behavior of the *X*^2^Π state as discussed in the paper of Laporta et al. (2016). An analysis of this aspect will be carried out in section Scaling Laws for Rapp and Briglia DEA Cross Section.

The aim of the present paper is to investigate the role of DEA process from vibrationally excited CO molecules in affecting the whole kinetics of reacting CO under conditions where appreciable concentrations of vibrationally excited states are present. These conditions can be found in the NRP atmospheric CO discharges with inter-pulse delay times *t*_*id*_ = 1 μ*s*, where memory effects along the different pulses (Pietanza et al., 2018a,b) can result in very excited vdf and eedf. Calculations for *t*_*id*_ = 25 μ*s* are also reported to be compared to the *t*_*id*_ = 1 μ*s* case.

For the present study, we select, between the different models, reported in Pietanza et al. (2018b), that one corresponding to optically thick plasmas with quenching processes, i.e., with the presence of the deactivation of the metastable *a*^3^Π state and consequent vibrational excitation of the vibrational manifold of CO.

The paper is divided into 6 sections. After the introduction, section The Model describes the model emphasizing the main differences with that one developed in Pietanza et al. (2018a,b), i.e., the inclusion of the DEA process for CO. Section Short Inter-Pulse Delay Time discusses the results for the short inter-pulse delay time case (*t*_*id*_ = 1 μ*s*), emphasizing the role of DEA in affecting macroscopic quantities, such as the molar fractions of the different species, including electrons, the electron and vibrational temperatures and the reactive channel rate coefficients, and microscopic quantities, such as vdf and eedf.

Section DEA Rate Coefficients reports the DEA rate coefficients under selected pulses, discussing the role of DEA from the complete set of cross sections involving the state *X*^2^Π (DEA(*X*^2^Π)), as compared with the *v* = 0 experimental contribution, DEA_RB_. Section Long Inter-pulse Delay Time reports results for a longer inter-pulse delay time case, i.e., *t*_*id*_ = 25 μ*s*. Section Scaling Laws for Rapp and Briglia DEA Cross Section considers a scaling law for the cross sections of DEA measured by Rapp and Briglia (1965) and their role in affecting the global results. Finally, section Conclusions reports conclusions and perspectives.

## The Model

The model is based on the solution of a time dependent Boltzmann equation for the calculation of the eedf, coupled to the non-equilibrium vibrational kinetics of CO molecules for the calculation of the vdf in the ground electronic state of CO and the electronic excited state kinetics of CO, C, and O species, as well as, with a simple dissociation-recombination and ionization-recombination kinetics describing the plasma mixture (Capitelli et al., 2016; Pietanza et al., 2017a,b, 2018a,b).

All the kinetics are self consistently and time dependent solved. Equations and details can be found in Pietanza et al. (2017a, 2018a,b).

The plasma mixture considered is composed by the following species: CO(*X*^1^Σ^+^, *v* = 1–80), CO_2_, C, O, CO^+^, ${\text{CO}}_{2}^{+}$, C^+^, O^+^, and e^−^. The energy level diagrams of CO, C and O are schematically represented in Figure 1 of Pietanza et al. (2018a).

Besides the ground state vibrational ladder, we consider several CO electronic excited states: three triplet states, *a*^3^Π (6.006 eV), *a*′3Σ^+^(6.863), *b*^3^Σ^+^(10.40 eV) and four singlet states, *A*^1^Π (8.03 eV), *B*^1^Σ^+^(10.78 eV), *C*^1^Σ^+^(11.40 eV), and *E*^1^Σ^+^(11.52 eV).

For C and O atoms, only four and five electronic levels, including the ground one, are accounted, namely C(^3^P), C(^1^D), C(^1^S), C(^5^S^0^) and O(^3^P), O(^1^D), O(^1^S), O(^3^S^0^) and O(^5^S^0^), while CO_2_, C^+^ and O^+^ are considered only in their ground states (see Figure 1b of Pietanza et al., 2018a).

The plasma chemistry model is the same presented in Pietanza et al. (2018a,b), but with the inclusion of the DEA process for CO. All the processes included into the model are listed in Table 1. In particular, CO dissociation can occur by direct electron impact mechanism (DEM), see process C_1_, and by pure vibrational excitation mechanism (PVM), see processes C_2_ (PVM_1_) and C_3_ (PVM_2_), involving all the vibrational ladder. Beside DEM process, also resonant electron impact dissociation (RES) process is included in the model, see process C_4_, in which dissociation is induced indirectly through the activation of the intermediate negative ion vibrational state *CO*^−^(^2^Π). The corresponding cross sections are generally lower than the direct ones (process C_1_), but dramatically increase with the vibrational quantum number, as in the case of DEA, being thus comparable to the DEM ones for higher vibrational levels.

**Table 1:** Plasma chemistry model.

**No**.	**Reaction**	**References**
C_1_	*e*+*CO*(*v*)↔*e*+*C*+*O*	Cosby, 1993
C_2_	*CO*(*v*)+*M*→*C*+*O*+*M*	Macdonald et al., 2016
C_3_	*CO*(*v*)+*CO*(*w*) → *CO*_2_+*C*	Gorse et al., 1984; Essenigh et al., 2006; Barreto et al., 2017
C_4_	*e*+*CO*(*v*) → *CO*^−^(*X*^2^Π) → *e*+*C*(^3^*P*)+*O*(^3^*P*)	Laporta et al., 2016
C_5_	*C*+*O*+*M*→*CO*(0)+*M*	Kozak and Bogaerts, 2014, 2015
C_6_	*e*+*CO*(*v*)↔*e*+*CO*^+^	Itikawa, 2015
C_7_	*e*+*C*(^3^*P*)↔*e*+*C*^+^	Wang et al., 2013
C_8_	*e*+*O*(^3^*P*)↔*e*+*O*^+^	Laher and Gilmor, 1990
C_9_	*CO*^+^+*e*→*C*+*O*	Kozak and Bogaerts, 2014, 2015
C_10_	*C*^+^+*e*→*C*	Pietanza et al., 2018a,b
C_11_	*O*^+^+*e*→*O*	Pietanza et al., 2018a,b
C_12_	*e*+*CO*(*X*^1^Σ^+^, *v*) → *CO*^−^(*X*^2^Π) → *C*(^3^*P*)+*O*^−^(^2^*P*)	Laporta et al., 2016
C_13_	*e*+*CO*(*X*^1^Σ^+^, *v* = 0) → *CO*^−^(*A*^2^Σ, ….) → *C*(^3^*P*)+*O*^−^(^2^*P*)	Rapp and Briglia, 1965; Itikawa, 2015
C_14_	*C*(^3^*P*)+*O*^−^(^2^*P*) → *e*+*CO*(*X*^1^Σ^+^, *v* = 0)	Fehsenfeld et al., 1966

The PVM_2_ process (C_3_) is called Boudouard or disproportioning reaction and the corresponding rate coefficient has been obtained by the equations used in Gorse et al. (1984), with an activation energy of 8.3 eV, recently calculated by Barreto et al. (2017). C and O recombination process forming CO molecules (C_5_) together with CO, C and O ionization (C_6_-C_8_) and CO^+^, C^+^, and O^+^ recombination processes (C_9_-C_11_) are also included into the model.

The explicit rate coefficient expressions of processes C_1_-C_11_ in Table 1 can be found in Pietanza et al. (2017a, 2018a,b).

In addition, in the present work, we include also DEA process for CO through the *X*^2^Π channel (DEA(*X*^2^Π)) from all the vibrational levels, see processes C_12_ in Table 1. The relevant vibrational state-resolved cross sections are provided by Laporta et al. (2016).

We insert also the experimental DEA process from *v* = 0, see process C_13_ in Table 1, whose cross section was reported by Rapp and Briglia (1965) and Itikawa (2015). This cross section should include the contribution of other resonant channels, as for example the *CO*^−^(*A*^2^Σ) state. Unfortunately, higher vibrational state cross sections of process C_13_ are not available up to now, however, due to the importance of the process, section Scaling Laws for Rapp and Briglia DEA Cross Section will discuss possible scaling laws, useful to extend the *v* = 0 cross section also to higher vibrational levels and the effect of inclusion of such cross sections in the kinetics.

As inverse process of C_12_ and C_13_, we include process C_14_, with a global rate coefficient of 5 10^−10^ cm^3^/s taken from Fehsenfeld et al. (1966).

The CO vdf is obtained from the corresponding vibrational master equations including the following e-V, V-V, V-T, SE and reactive contribution (see Pietanza et al., 2018a,b).

(2) $$\begin{array}{l} \frac{dN_{v}}{dt}={\left(\frac{dN_{v}}{dt}\right)}_{e-V}+{\left(\frac{dN_{v}}{dt}\right)}_{V-V}+{\left(\frac{dN_{v}}{dt}\right)}_{V-T}\\ +{\left(\frac{dN_{v}}{dt}\right)}_{SE}+{\left(\frac{dN_{v}}{dt}\right)}_{React} \end{array}$$

The e-V (electron-vibration) term describes the energy exchange between electrons and the CO vibrational ladder. A complete set of resonant e-V cross sections involving all the CO vibrational ladder has been provided by Laporta et al. (2012). The V-V, V-T, SE terms correspond to vibrational energy exchange processes due to vibration-vibration (V-V), vibration-translation (V-T), and spontaneous emission (SE). Finally, the last term describes the reactive channel contribution due to the dissociation-recombination (C_1_-C_5_) and ionization-recombination processes, involving the CO vibrational ladder (C_6_) reported in Table 1. This last term includes also the contribution of the quenching of the metastable *a*^3^Π state of CO, which is assumed to pump energy into the level *v* = 27

(3) $$\begin{array}{c} CO\left(a^{3}\Pi ,v=0\right)+CO\to CO\left(X^{1}\Sigma^{+},v=27\right)+CO \end{array}$$

This process, which has an essential role in modifying the vdf, is included with an upper limit rate coefficient of 1.21 10^−10^ cm^3^/s.

The electronic excited state kinetics of CO, O, and C atoms, instead, is described by the following differential equation in which the terms due to electron impact excitation and de-excitation, spontaneous emission and quenching processes are accounted, i.e.,

(4) $$\begin{array}{c} \frac{dn_{i}}{dt}=K_{exc}^{i}n_{e}n_{0}-K_{de-exc}^{i}n_{e}n_{i}-\sum_{j<i}\lambda_{ij}A_{ij}n_{i}-Q \end{array}$$

where *n*_*i*_ is the population density of the *i*^th^ electronic state, $K_{exc}^{i}$ and $K_{de-exc}^{i}$ the electron impact excitation (from ground) and de-excitation rate coefficients, n_e_ and n_0_ the electron and ground state densities, λ_*ij*_ the escape factor and *A*_*ij*_ the Einstein coefficient of spontaneous emission toward lower electronic states j. $K_{exc}^{i}$ and $K_{de-exc}^{i}$ rate coefficients are calculated by integrating the instantaneous eedf over the corresponding electron impact cross sections, taken from the Itikawa database for CO (Itikawa, 2015), from Laher and Gilmor for O (Laher and Gilmor, 1990) and Wang et al. for C (Wang et al., 2013). In the present paper, we consider an optically thick plasma so that λ_*ij*_ = 0 for all considered optical transitions involving the electronic excited states. The Q term includes all the quenching processes, the most important for CO is that one in equation (3). Also some other quenching processes for C and O electronic states are included in the model as reported in Pietanza et al. (2018a,b).

## Results: General Considerations

In this section, we report results for a NRP discharge sustained by a sequence of modulated electric field pulses with a pulse duration *t*_p_ = 20 ns and an inter-pulse delay time *t*_*id*_ = 1 μ*s* (sections Short Inter-pulse Delay Time and DEA Rate Coefficients), while the results with *t*_*id*_ = 25 μ*s* are presented in section Long Inter-Pulse Delay Time.

**Figure 1:**
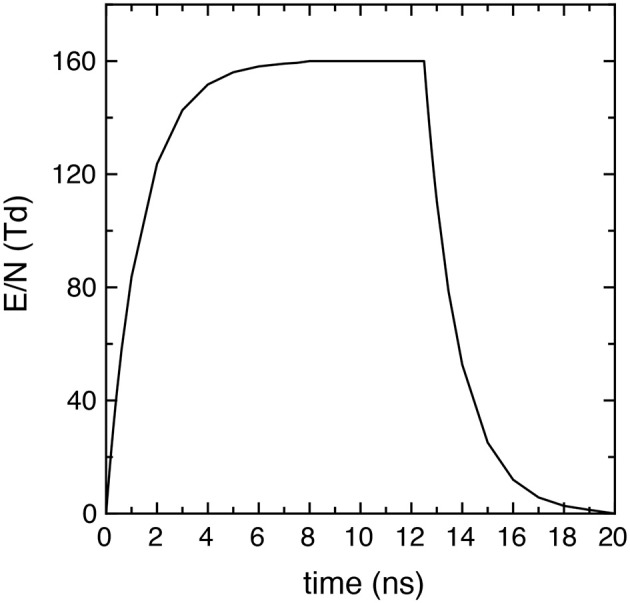
Reduced electric field time profile in each pulse used in all the simulations (E_M_/N = 160 Td, *t*_r_ = *t*_f_ = 7.5 ns, τ_*r*_ = τ_*f*_ = 1.35 *ns* and *t*_p_ = 20 ns).

The electric field is characterized by a time-dependent profile (Pietanza et al., 2018a,b) described by the following analytical expression

(5) $$\begin{array}{c} E\left(t\right)=\{\begin{array}{cc} E_{M}\left(1-e^{-\;\frac{t}{\tau_{r}}}\right) & t\in \left[0,t_{r}\right)\\ E_{M} & t\in \left[t_{r},t_{p}-t_{f}\right)\\ E_{M}e^{-\frac{t}{\tau_{f}}} & t\in \left[t_{p}-t_{f},t_{p}\right)\\ 0 & t\in \left[t_{p},t_{pd}\right] \end{array} \end{array}$$

where E_M_ is the peak intensity, *t*_r_ and *t*_f_ the rise and fall times and their characteristic times τ_*r*_ and τ_*f*_, *t*_p_ the pulse and *t*_pd_ the post-discharge duration. Successive pulses are separated by an inter-pulse delay time *t*_id_ = *t*_p_ + *t*_pd_. In particular, in the simulations E_M_/N = 160 Td, *t*_r_ = *t*_f_ = 7.5 ns, τ_*r*_ = τ_*f*_ = 1.35 *ns*, where *N* is the total number density (cm^−3^). Figure 1 reports the time behavior of the applied reduced electric field E/N (E/N = 0 in the afterglow) in one pulse (20 ns), showing that it presents a maximum value of 160 Td in the time interval [7.5 ns, 12.5 ns].

In the short inter-pulse delay time case (*t*_*id*_ = 1 μ*s*), we limit our discussion to the first 4 pulses and corresponding afterglows, while in the long inter-pulse delay time case (*t*_*id*_ = 25 μ*s*), we consider 20 pulses and corresponding afterglows.

In both cases, we consider an atmospheric (*P* = 1 atm) CO plasma at constant gas temperature (*T*_g_ = 1,000 K) and, as initial condition, we fix a Boltzmann distribution of the vibrational levels at *T*_v_ (*t* = 0) = *T*_g_ and a Maxwell eedf at *T*_e_(*t* = 0) = *T*_g_. The initial molar fractions of the considered species are about 1 for CO, 10^−6^ for electrons and negligible values for the other considered species.

### Short Inter-Pulse Delay Time

In this section, we analyze the effect of introducing DEA process in a short inter-pulse delay time case study, i.e., *t*_*id*_ = 1 μ*s*. In general, the results with the insertion of DEA from all vibrational levels qualitatively follow those described in Pietanza et al. (2018a,b), presenting, however, for the considered case study, no-negligible differences with the progression of the considered pulses. This point will appear clear in the following.

**Figure 2:**
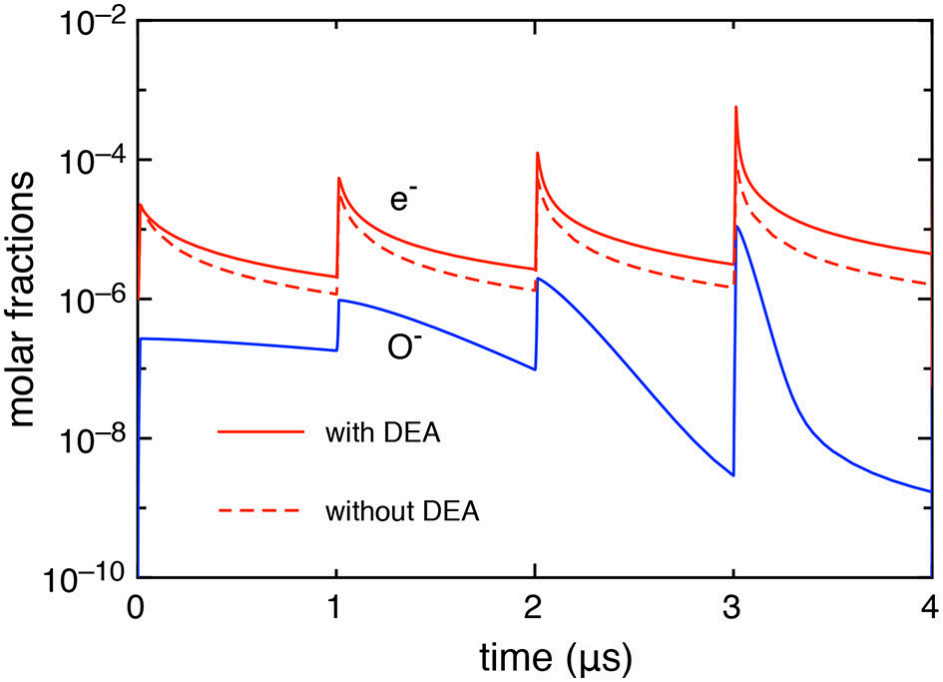
Time evolution of electron and O^−^ molar fractions (*t*_*id*_ = 1 μ*s*).

First, we report, in Figure 2, the electron and O^−^ molar fractions as a function of the time when the DEA processes are included and the corresponding electron molar fraction when the DEA processes are neglected.

The differences in the electron molar fraction in the two cases increase with the pulses: at the last pulse, the electron molar fraction, at the maximum of the discharge, is 5.05 10^−4^ with DEA and 9.0 10^−5^ without DEA, while, at the end of the post-discharge, 4.45 10^−6^ with DEA and 1.6 10^−6^ without DEA. The increase of electron density when DEA is inserted in the kinetics is due to the effect of the global associative attachment (reaction C_14_ in Table 1).

**Figure 3:**
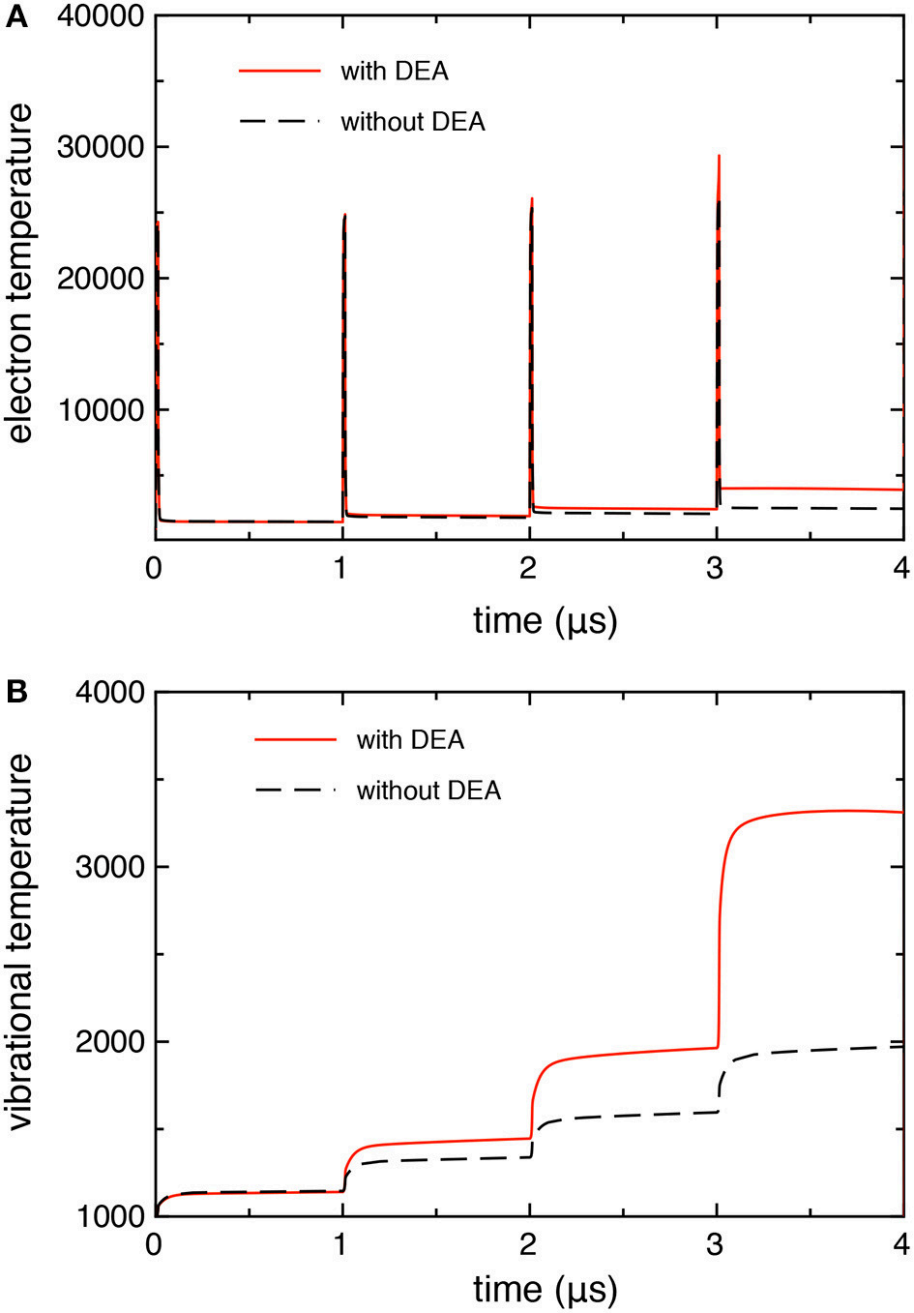
**(A)** Electron, and **(B)** vibrational temperature as a function of time, calculated with and without DEA processes (*t*_*id*_ = 1 μ*s*).

Figures 3A,B compares the time evolution of electron temperature (from the average electron energy) and the 0–1 vibrational temperature calculated with and without the DEA processes. In both quantities, we observe larger values when taking into account the DEA processes due to the corresponding behavior of electron molar fraction as reported in Figure 2.

**Figure 4:**
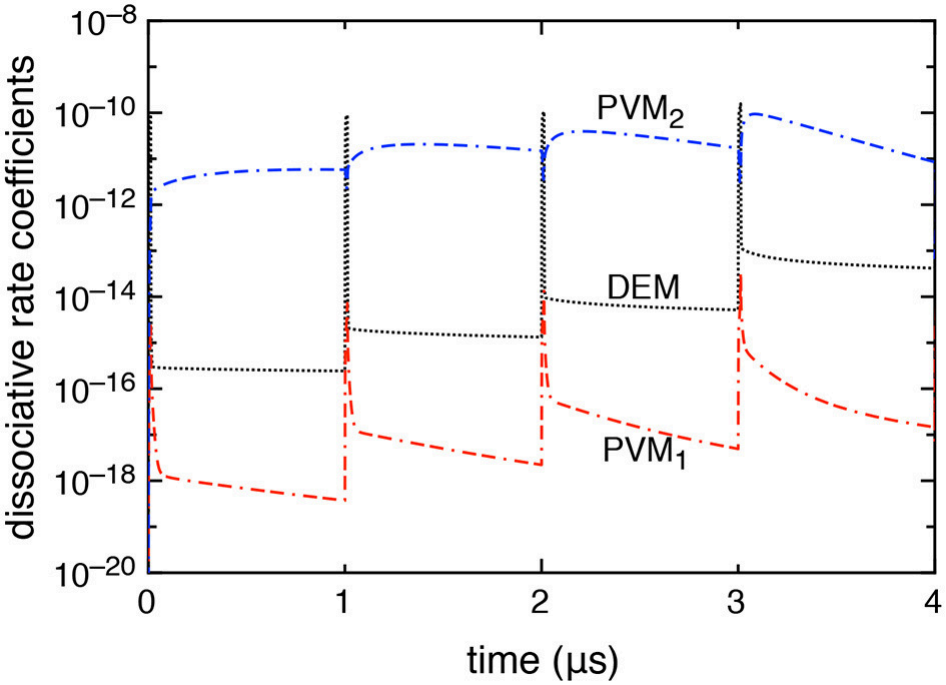
Dissociative rate coefficients by direct electron impact (DEM) and pure vibrational mechanism (PVM_1_, PVM_2_) as a function of time when DEA processes are included into the model (*t*_*id*_ = 1 μ*s*).

Figure 4 reports the time evolution of the dissociation rate coefficients by electron impact (DEM) and by pure vibrational mechanism (PVM_1_ and PVM_2_) in the case in which DEA processes are included. We can note that the DEM rate coefficient slightly prevails on the Boudouard one (i.e., the PVM_2_ one) under discharge conditions becoming less important in the corresponding afterglows. Actually, during the discharge, the electron density reaches its maximum peak value strongly increasing all electron impact processes. During the afterglow, the decrease of electron density and the presence of excited vdf makes the dissociation process induced by vibrational excitation, in particular the PVM_2_ mechanism, prevail over the others.

**Figure 5:**
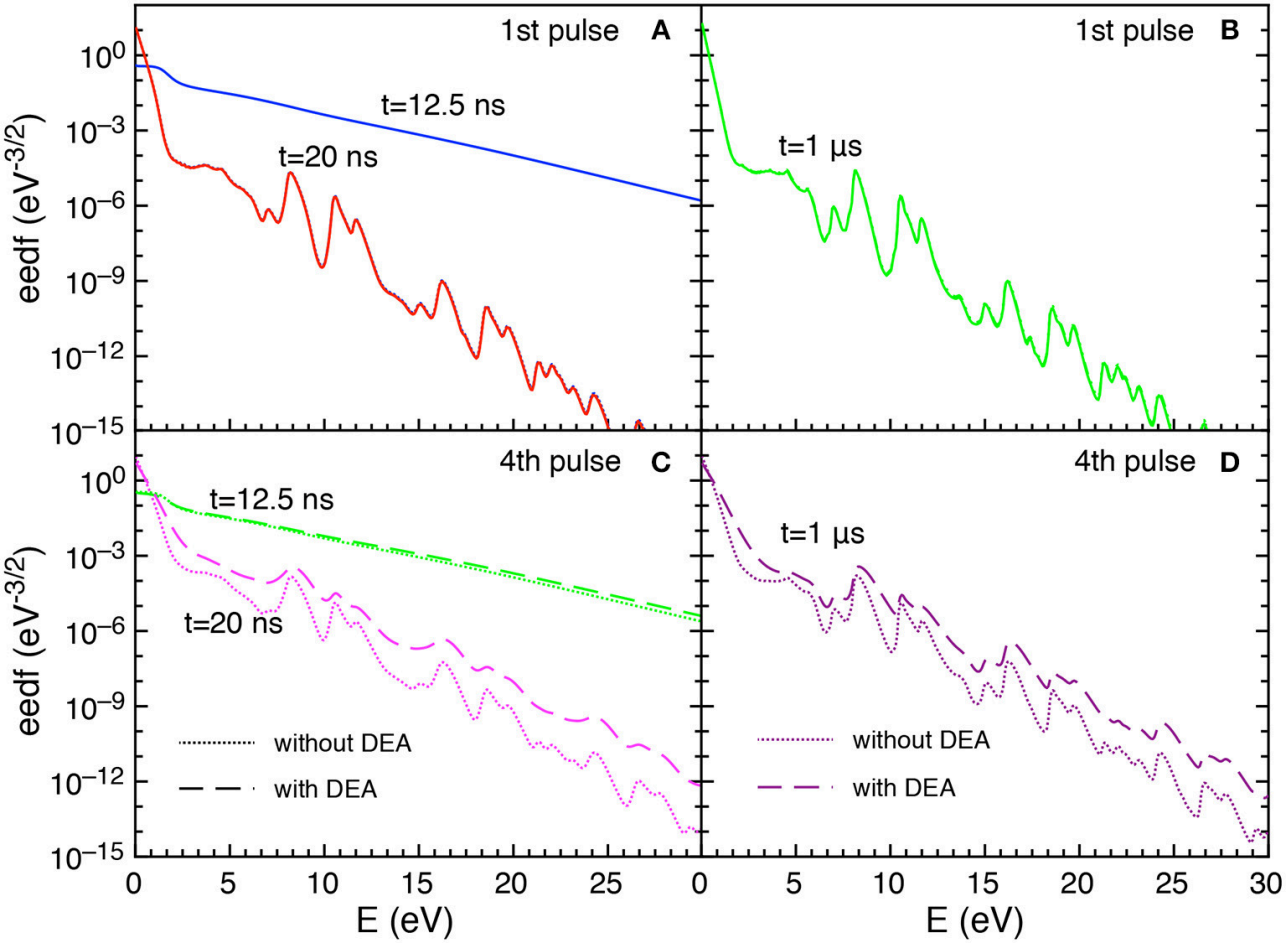
**(A–D)** Eedf as a function of time for selected pulses (1st and 4th) at selected times *t* = 12.5 ns, *t* = 20 ns and t = 1 μs with and without DEA (*t*_*id*_ = 1 μ*s*).

Let us now examine the trend of the eedf calculated with and without DEA. Figures 5A–D reports the eedf for selected pulses (1st and 4th) at different times during the discharge (*t* = 12.5 and *t* = 20 ns) and at the end of corresponding afterglows (*t* = 1 μs).

In particular, during the first pulse discharge and afterglow, the eedf plots with and without DEA are coincident, i.e., no role is exercised by DEA in affecting eedf. For both discharge and post discharge conditions of the first pulse, a well-structured eedf appears due to superelastic electronic collisions considered in the kinetics, as discusses in Pietanza et al. (2018a,b).

In the fourth pulse, the differences between DEA and no-DEA eedf is negligible at 12.5 ns while it becomes important at the end of pulse (*t* = 20 ns) and at the end of the post-discharge (*t* = 1μs). This behavior follows the dependence of eedf on the time evolution of either E/N and the vibrational temperature.

**Figure 6:**
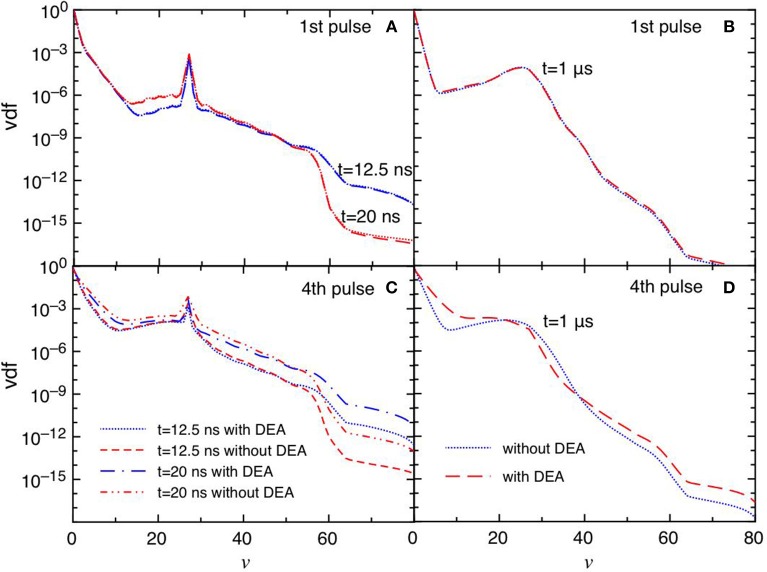
**(A–D)** Vdf as a function of time for selected pulses (1st and 4th) at selected times *t* = 12.5 ns, *t* = 20 ns and *t* = 1 μs with and without DEA (*t*_*id*_ = 1 μ*s*).

Figures 6A–D report the trend of vdf for the same conditions reported in Figure 5. The differences between the DEA and the no-DEA results are absent in the first pulse (Figures 6A,B), becoming important in the fourth pulse and corresponding afterglow (Figures 6C,D), following the eedf's behavior. It is evident the effect of the quenching process of the CO(*a*^3^Π) state [see equation (3)], which pumps vibrational energy in the ground state at *v* = 27 affecting the corresponding vdf either in discharge and post discharge conditions. In the last considered pulse, a redistribution of vibrational quanta over the whole vibrational ladder in both discharge and post discharge conditions is observed. This redistribution is due to e-V processes under discharge conditions (large ionization degree), and to V-V up pumping mechanism under post-discharge conditions. The excited vibrational distributions shown in Figure 6 are responsible of the increase of DEA rate coefficients as it will be discussed in the following sections.

### DEA Rate Coefficients

In this section, we want to emphasize the role of vibrational excitation in enhancing the total DEA rate coefficients.

**Figure 7:**
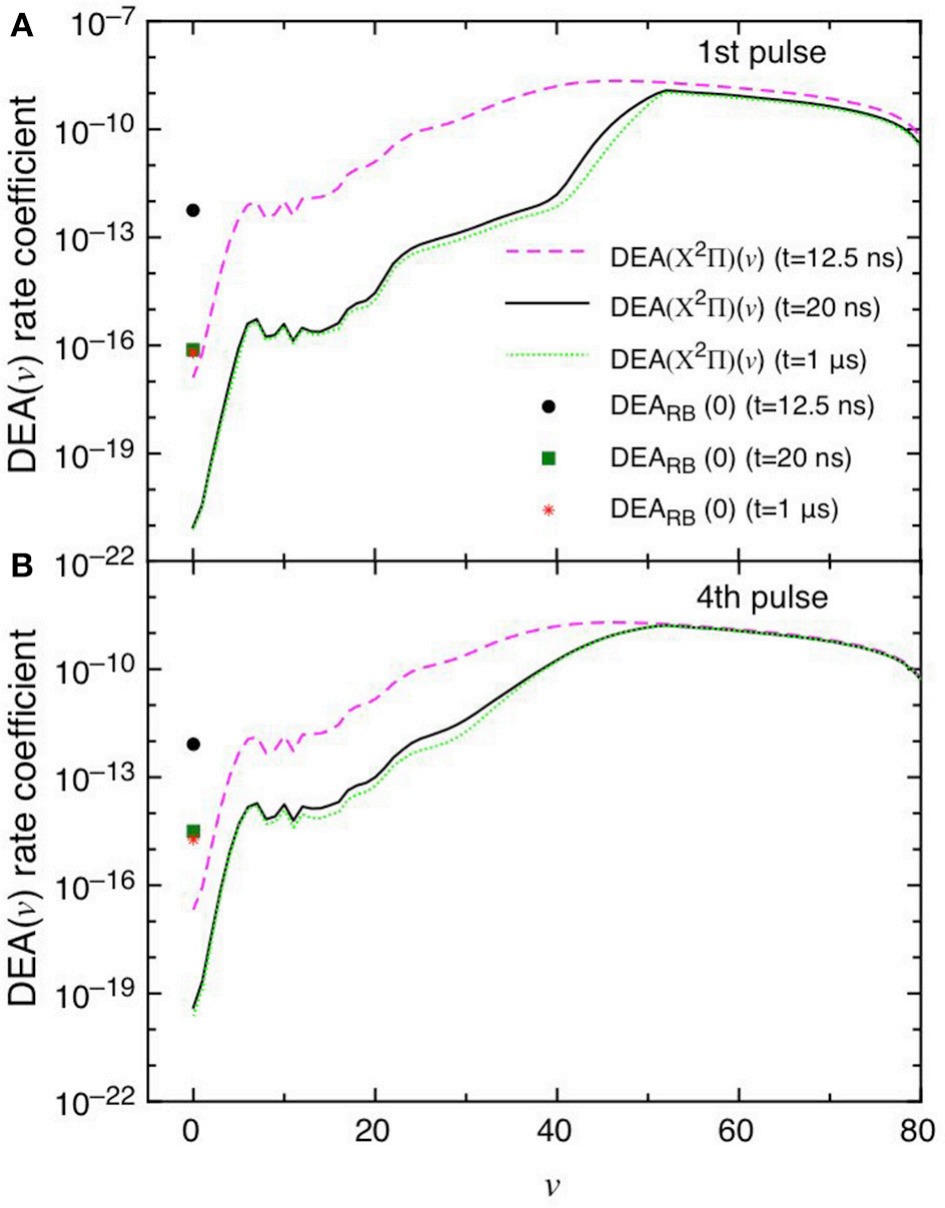
**(A,B)** Dissociative electron attachment rate coefficients DEA(X^2^Π)(*v*) as a function of *v* and DEA_RB_(0) derived from the experimental *v* = 0 Rapp and Briglia cross section at different pulses (1st and 4th) and different times (*t* = 12.5 ns, *t* = 20 ns and *t* = 1 μs).

**Figure 8:**
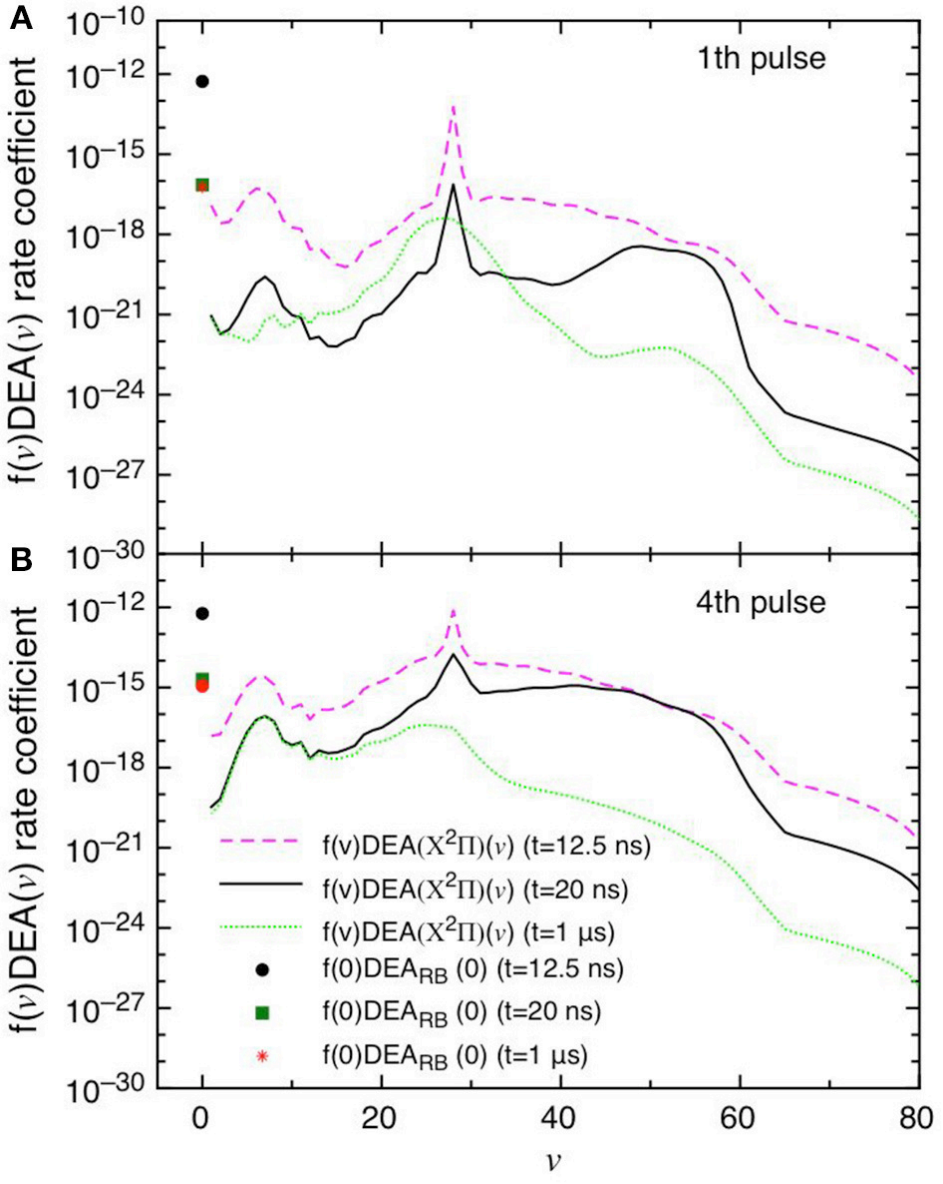
**(A,B)** Dissociative electron attachment rate coefficients modulated with the actual molar fractions of vibrational levels (f(*v*)DEA(X^2^Π)(*v*)) and f(0)DEA_RB_(0) at different pulses (1st and 4th) and different times (*t* = 12.5 ns, *t* = 20 ns and *t* = 1 μs).

This point can be better understood by looking to the dependence of DEA(*v*) rate coefficients as a function of vibrational quantum number reported in Figures 7A,B as well as their partial contributions, i.e. f(*v*)DEA(*v*), reported in Figures 8A,B for the selected two pulses, where f(*v*) represents the molar fraction of the *v*th vibrational state. Figures 7A,B, in particular, shows the calculated DEA(X^2^Π)(*v*) rate coefficients as a function of vibrational quantum number *v* as well as the DEA_RB_(0) rate coefficients calculated from the *v* = 0 experimental Rapp and Briglia cross section. In general, the DEA(X^2^Π)(*v*) rate coefficients overcome the DEA_RB_(0) one in a vast range (5 < *v* < 80) of the vibrational quantum number, independently of the considered pulse. The situation changes when multiplying the DEA rates for f(*v*) (Figures 8A,B). Inspection of the figure shows the increasing importance with the sequence of the pulses of the f(*v*)DEA(X^2^Π)(*v*) contribution as compared with the f(0)DEA_RB_(0) one, following the form of the reported vdf in Figures 6A–D.

In the first pulse, f(0)DEA_RB_(0) is larger than f(*v*)DEA(X^2^Π) in the whole *v* range at the maximum of E/N value (*t* = 12.5 ns) and also at *t* = 20 ns and in the post-discharge (*t* = 1 μs). In the last pulse, instead, f(*v*)DEA(X^2^Π)(*v*) overcome f(0)DEA_RB_(0) in a vast range of *v* especially at the end of the pulse, i.e., 20 ns.

**Figure 9:**
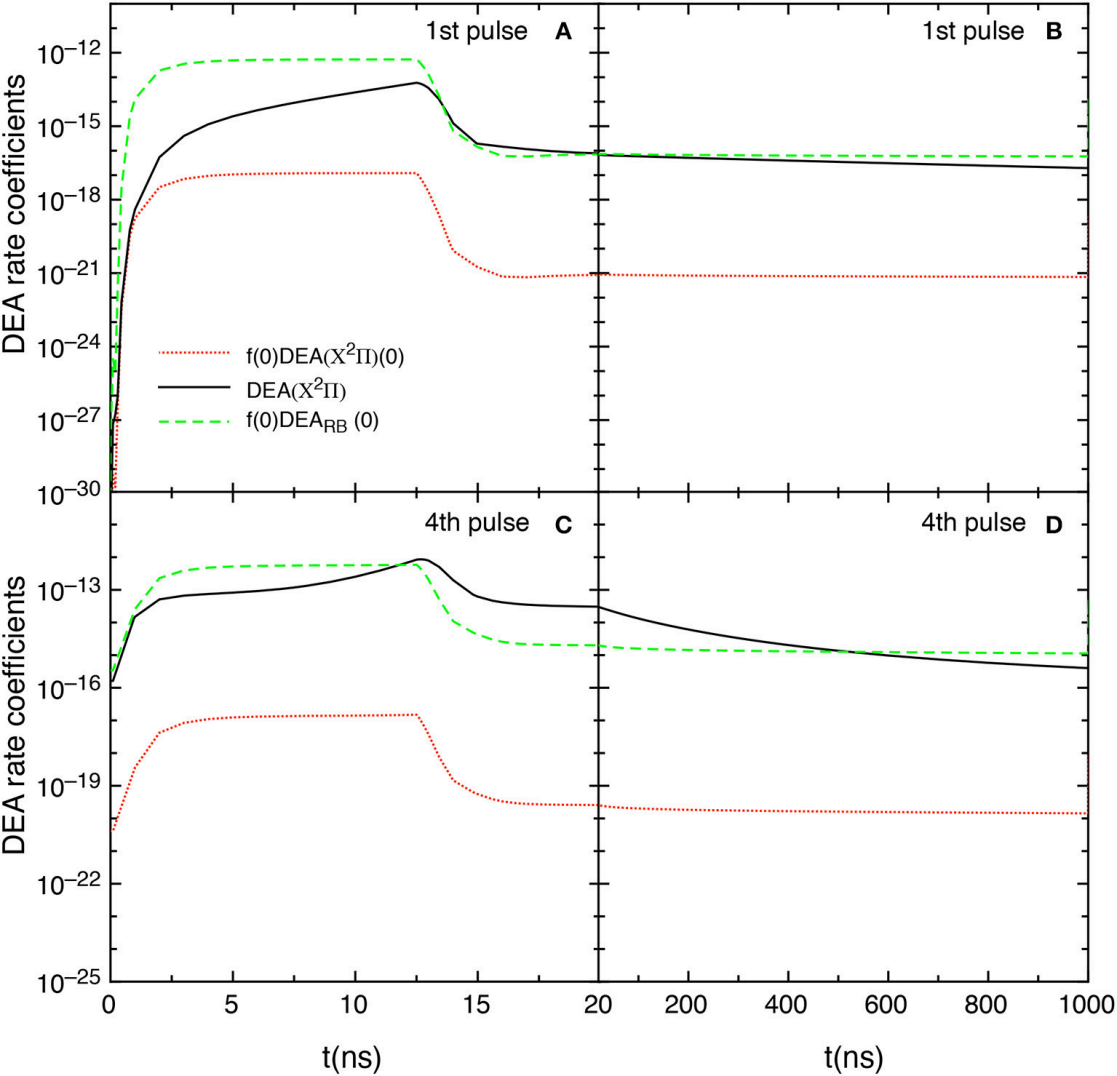
**(A–D)** Temporal evolution of the different DEA rate coefficients for the 1st and 4th pulses during discharge **(A,C)** and corresponding afterglows **(B,D)** (*t*_*id*_ = 1 μ*s*).

The competition between the different DEA channels are evidenced in Figures 9A–D which shows the behavior of the DEA rate coefficients as a function of the time for the first and fourth pulses and afterglows. The contribution labeled as DEA(X^2^Π) is calculated by

(6) $$\begin{array}{c} DEA\left(X^{2}\Pi \right)=\sum_{v}f(v)DEA\left(X^{2}\Pi \right)(v) \end{array}$$

In the first pulse, during the discharge regime, f(0)DEA_RB_(0) is larger than DEA(X^2^Π) until 12.5 ns, becoming very similar from 12.5 to 20 ns. The two main contributions are competitive in the post discharge regime (Figure 9B). In both situations, f(0)DEA(X^2^Π)(0) is orders of magnitude lower.

The situation reported for the fourth pulse (discharge regime) reduces the differences between f(0)DEA_RB_(0) and DEA(X^2^Π) until 12.5 ns inverting the situation from 12.5 to 20 ns. In the post discharge regime DEA(X^2^Π) > f(0)DEA_RB_(0) until 400 ns, the two terms appearing similar for *t* > 500 ns.

## Long Inter-Pulse Delay Time

**Figure 10:**
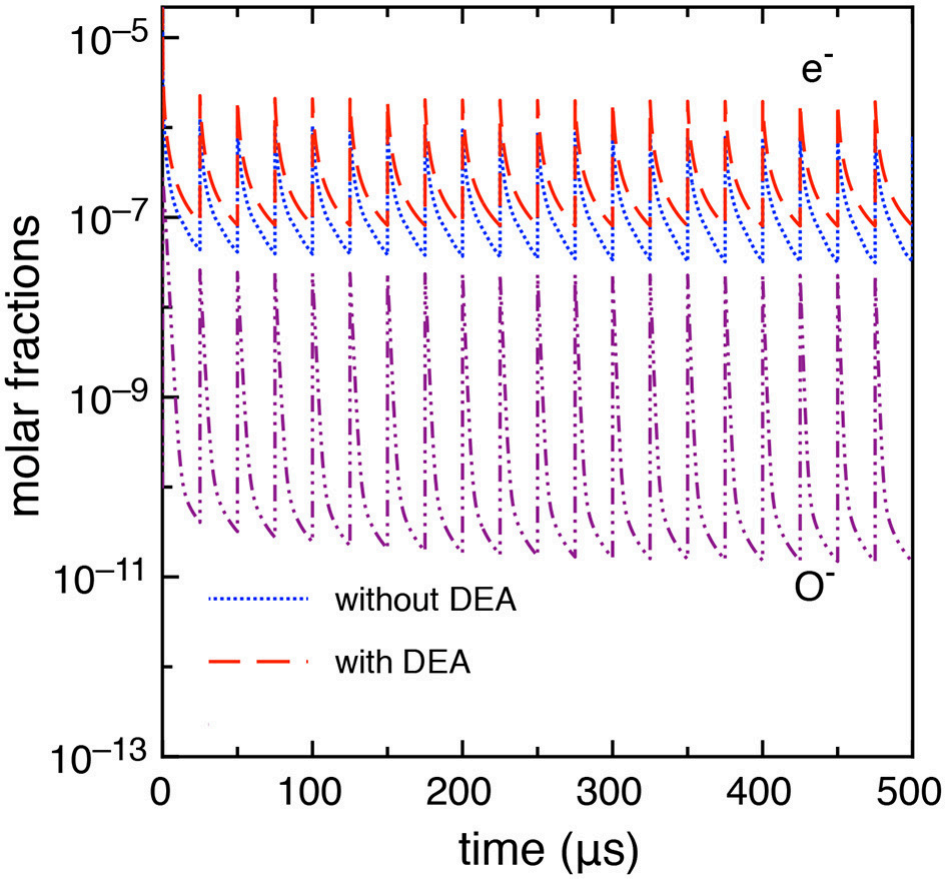
Time evolution of electron and O^−^ molar fractions (*t*_*id*_ = 25 μ*s*).

This case study differs from the previous one only by the inter-pulse delay time which is longer, i.e., *t*_*id*_ = 25 μ*s*. A more stable behavior is observed in this case as discussed in Pietanza et al. (2018a,b) resulting in a quasi-stable sequence of pulses and afterglows with a small dependence of the results on the DEA processes. Figure 10 reports the electron molar fraction calculated with and without DEA up to the 20th pulse. In the same figure, we report the molar fraction O^−^. The differences, even though not negligible, are much smaller than the previous *t*_*id*_ = 1 μ*s* case. Moreover, as it can be seen from Figure 10, the electron molar fraction is of the order of 10^−5^ not able to promote the role of vibrational excited states in the whole kinetics.

**Figure 11:**
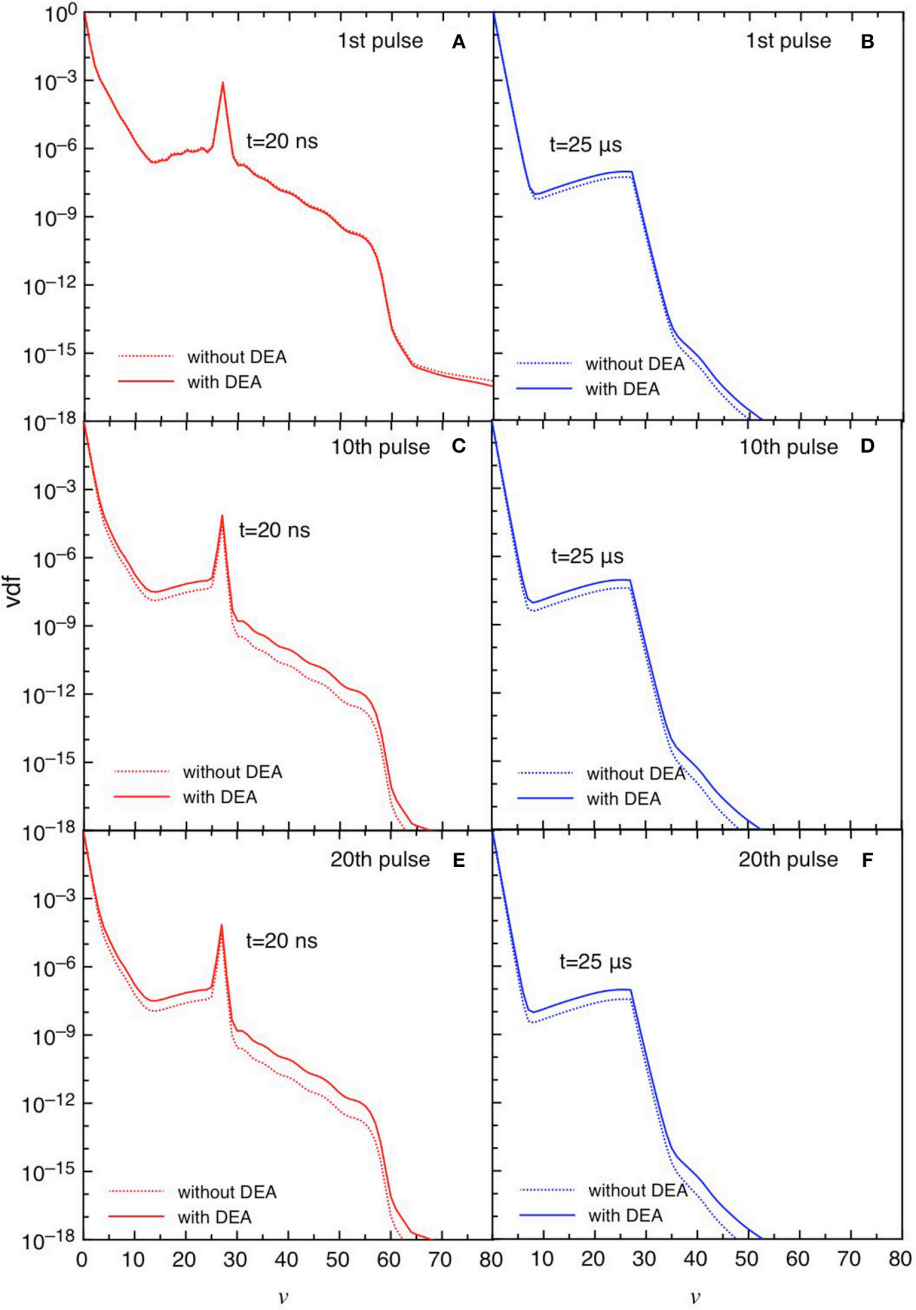
**(A-F)** Vdf at selected pulses (1st, 10th, and 20th) at the end of the discharge (*t* = 20 ns) **(A,C,E)** and post-discharge (*t* = 25 μs) **(B,D,F)** (*t*_*id*_ = 25 μ*s*).

**Figure 12:**
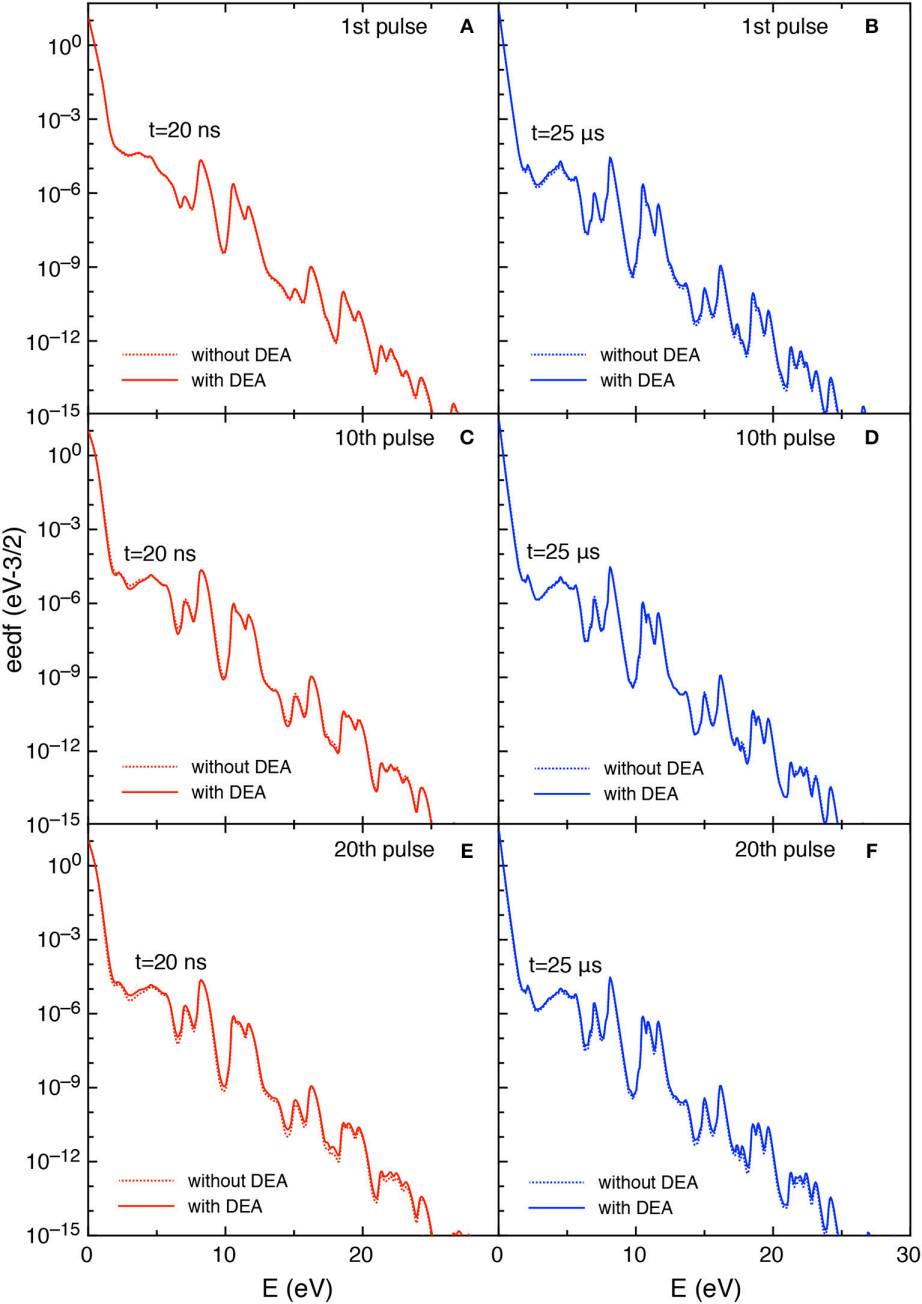
**(A-F)** Eedf at selected pulses (1st, 10th, and 20th) and at selected times in discharge (*t* = 20 ns) **(A,C,E)** and post-discharge (*t* = 25 μs) **(B,D,F)** (*t*_*id*_ = 25 μ*s*).

Figures 11, 12 report the vdf and the eedf at the three selected pulses, at the end of the discharge (*t* = 20 ns) and of the post-discharge (*t* = 25 μ*s*). Their time evolution in each pulse repeat themselves without the memory effects observed in the *t*_*id*_ = 1 μ*s* case. Moreover, the insertion of DEA processes has a smaller influence as compared with the corresponding results in the *t*_*id*_ = 1 μ*s*, especially for the eedf.

**Figure 13:**
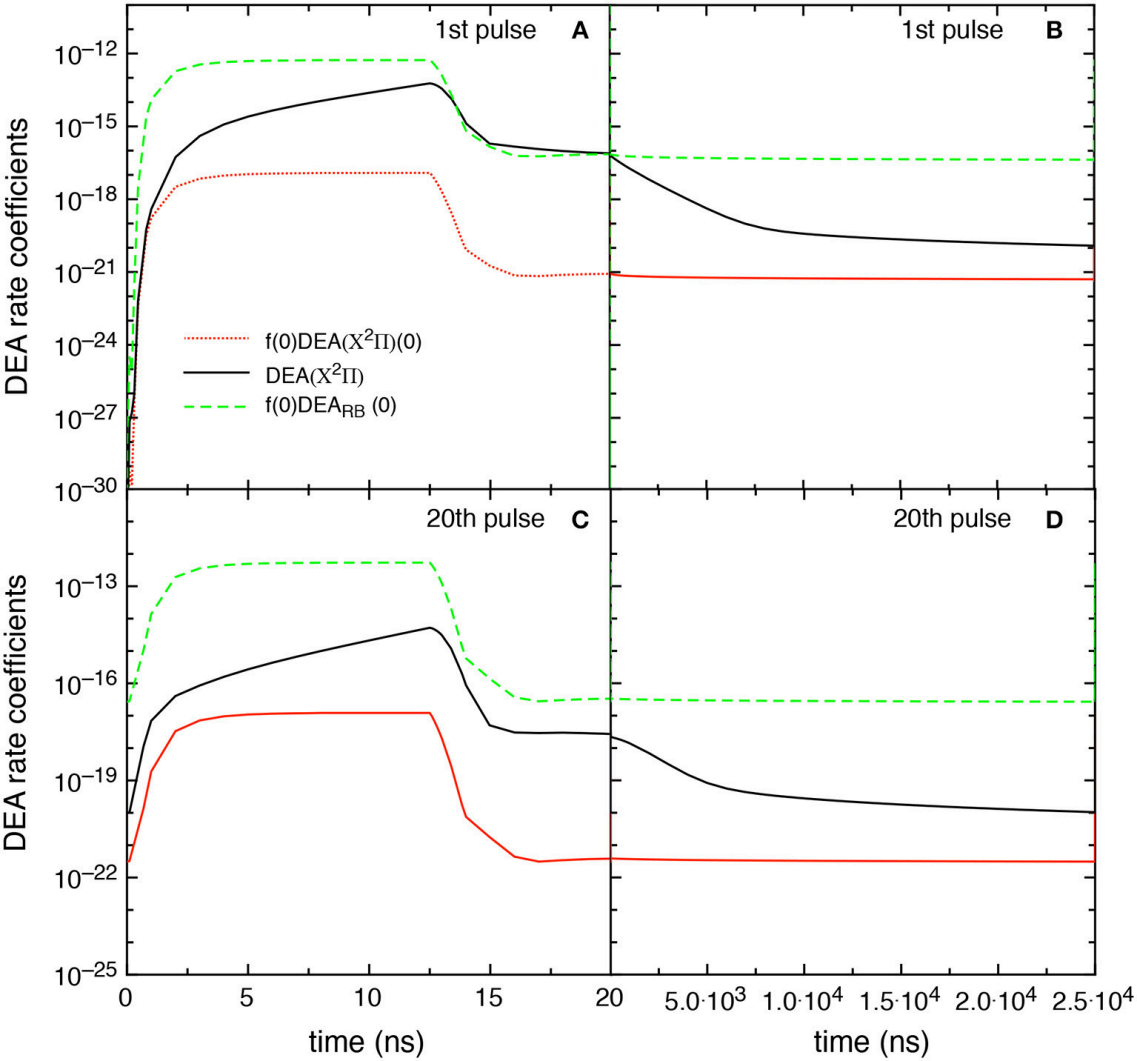
Temporal evolution of the different DEA rate coefficients for the 1st and 20th pulses during discharge **(A,C)** and corresponding afterglows **(B,D)** (*t*_*id*_ = 25 μ*s*).

Figure 13 reports the different DEA rate coefficients, i.e., f(0)DEA_RB_(0), DEA(X^2^Π), and f(0)DEA(X^2^Π)(0), as a function of time in discharge and post discharge conditions for the first and the 20th pulse, in the *t*_*id*_ = 25 μ*s* case. Qualitatively, the results follow those reported in Figure 9 even though the DEA(X^2^Π) contribution decreases its importance due to the presence of less pumped vibrational distributions. It is worth noting the no-time dependence of f(0)DEA_RB_(0) contribution in the post discharge for both pulses compared with the strong decay of DEA(X^2^Π). The behavior of f(0)DEA_RB_(0) is controlled by the form of the eedf strongly influenced by the superelastic electronic collisions, while the decay of DEA(X^2^Π) is controlled by the corresponding decay of vdf.

## Scaling Laws For Rapp and Briglia DEA Cross Section

As already underlined, vibrational-state resolved DEA cross sections are available only for the X^2^Π channel (Laporta et al., 2016) and no data do exist for the dependence on *v* of the experimental *v* = 0 cross section of Rapp and Briglia (1965).

Due to the importance of the latter cross section, the insertion of the corresponding vibrational state dependence could have an impact on the kinetics results. In this section, we discuss such impact by making reasonable scaling law assumptions on the *v*-dependence of the experimental DEA_RB_ cross section.

As a first hypothesis, we can use the same *v*-dependence of the X^2^Π channel cross section, i.e., by applying

(7) $$\begin{array}{c} \sigma_{v>0}^{RB}=\sigma_{v>0}^{L}{\left(\frac{\sigma_{0}^{RB}}{\sigma_{0}^{L}}\right)}^{MAX} \end{array}$$

where $\sigma_{v>0}^{RB}$ and $\sigma_{v>0}^{L}$ are, respectively, the DEA_RB_(*v*) and the DEA(X^2^Π)(*v*) cross section of the *v*th vibrational level and $\sigma_{0\;MAX}^{RB}$ and $\sigma_{0\;MAX}^{L}$ the corresponding maximum value of the *v* = 0 cross section.

However, such scaling law predicts too high cross section values which go beyond the reasonable limit of the rigid sphere model, i.e., by supposing a maximum radius of 3 Å at *v* = 80, $\pi a_{0}^{2}\approx 30$ Å^2^. For this reason, the following reduced scaling law can be used to limit the increase of the cross section for high *v* levels, with *n* a parameter which can assume positive integer or fractional values

(8) $$\begin{array}{c} \sigma_{v>0}^{RB}=\frac{\sigma_{v}^{L}}{v^{n}}{\left(\frac{\sigma_{0}^{RB}}{\sigma_{0}^{L}}\right)}^{MAX} \end{array}$$

**Figure 14:**
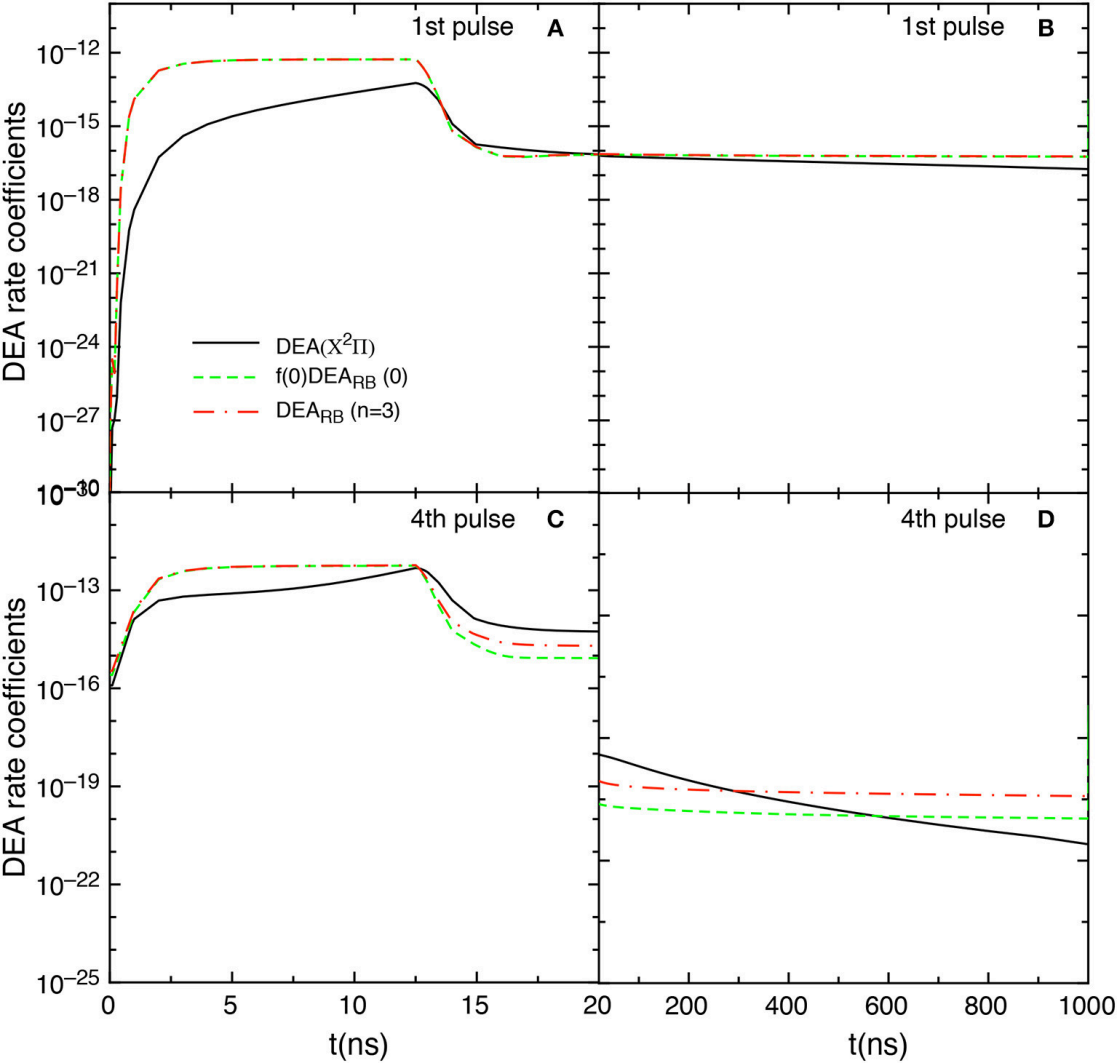
Temporal evolution of f(0)DEA_RB_(0), DEA_RB_(*n* = 3) and DEA(X^2^Π) contributions for the 1st and the 4th pulses during discharge **(A,C)** and corresponding afterglows **(B,D)** in the *t*_*id*_ = 1 μ*s* case when scaled cross sections are included.

Let us now examine the effect on the kinetics of the DEA_RB_ scaled cross sections obtained according equation (8) with *n* = 3. Figure 14 compares the f(0)DEA_RB_(0), the DEA(X^2^Π) and the DEA_RB_(*n* = 3) rate coefficient contributions obtained by including the scaled cross sections in the *t*_id_ = 1 μs test case. The DEA_RB_(*n* = 3) contribution is calculated by

(9) $$\begin{array}{c} DEA_{RB}\left(n=3\right)=\sum_{v}f(v)DEA_{RB}^{scaled\_n=3}(v) \end{array}$$

where $DEA_{RB}^{scaled\text{\_}n=3}\left(v\right)$ are the corresponding DEA_RB_ rate coefficients calculated from the scaled cross sections of equation (8) with *n* = 3.

The insertion of such cross sections shows results very similar to those obtained by including only the *v* = 0 contribution, i.e., the f(0)DEA_RB_(0) and DEA_RB_(*n* = 3) contributions are essentially equal in the first pulse. Differences occur in the last considered pulse (4th) for *t* > 12 ns as well as in the post discharge regime.

## Conclusions

The introduction of DEA from vibrationally excited states of CO plays an important role in NRP CO discharges with an inter-pulse delay times *t*_id_ = 1 μs having a minor role with *t*_id_ = 25 μs. The bulk of results have been obtained by inserting in the global kinetic model, described in Pietanza et al. (2018a,b), the DEA process through the resonant state *X*^2^Π characterized by *v*-state resolved cross sections

(10) $$\begin{array}{c} e+CO\left(X^{1}\Sigma^{+},v\right)\to CO^{-}\left(X^{2}\Pi \right)\to C{(}^{3}3P)+O^{-}{(}^{2}P) \end{array}$$

and the experimental DEA cross section, which should include the effect of all the other resonant state, i.e., *A*^2^Σ, ….

(11) $$\begin{array}{l} e+CO\left(X^{1}\Sigma^{+},v=0\right)\to CO^{-}\left(A^{2}\Sigma ,\ldots .\right)\to C{\text{(}}^{3}P)\\ +O^{-}{(}^{2}P) \end{array}$$

for which we do not have the dependence on the vibrational quantum number.

A scaling law has been considered obtaining a complete set of cross sections for the transition described by equation (11). Insertion of this new set of cross sections on the kinetics shows results qualitatively in line with the bulk of results obtained by inserting only the *v* = 0 contribution, showing however some differences especially in the last considered pulses, when important vibrationally excited vdf are achieved. However, future work in this direction is necessary to better characterize the dissociative cross sections for all resonant states beyond the contribution of *CO*^−^(*X*^2^Π).

Another important point to be better investigated is the characterization of the process

(12) $$\begin{array}{c} C+O^{-}\to e^{-}+CO\left(X^{1}\Sigma^{+},v=0\right) \end{array}$$

which produces a source of extra-electrons, becoming important to form extended vibrational distributions able to increase the DEA process. This point should be better quantified by considering the inverse reaction as populating the different vibrational levels, i.e.,

(13) $$\begin{array}{c} C+O^{-}\to e^{-}+CO\left(X^{1}\Sigma^{+},v>0\right) \end{array}$$

A perspective of this work will be the insertion of the CO reacting kinetics developed in the present work, as well as in Pietanza et al. (2018a,b) and Pietanza et al. (2017a,b), in a complex model for the activation of CO_2_ under non-equilibrium plasmas (Kozak and Bogaerts, 2014, 2015; Pietanza et al., 2016a,b, 2017c; Belov et al., 2017; Bogaerts et al., 2017a,b; Capitelli et al., 2017; Klarenaar et al., 2017; Silva et al., 2018). At the moment, the existing data of the dissociative electron attachment of CO_2_ include only the global process in the cold gas approximation (i.e., the different vibrational ladders are in the ground state). No theoretical data with the present accuracy for CO do exist and probably one could use the present CO dissociative attachment cross sections to find a scaling law for CO_2_.

The insertion of the complicated kinetics of CO in the corresponding kinetics of CO_2_ will elucidate the role of CO processes in affecting eedf and vdf of the reacting CO_2_ mixture when the dissociation of CO_2_ is larger than 10%. In doing so one should also try to develop simplified models able to reduce the number of components as well as to insert analytical forms of vdf for describing the actual vibrational distributions of the different components (Colonna et al., 1999, 2006; Grofulovic et al., 2018; Macdonald et al., 2018).

## Data Availability

Publicly available datasets were analyzed in this study. This data can be found here: https://fr.lxcat.net/data/set_type.php.

## Author Contributions

All authors listed have made a substantial, direct and intellectual contribution to the work, and approved it for publication.

### Conflict of Interest Statement

The authors declare that the research was conducted in the absence of any commercial or financial relationships that could be construed as a potential conflict of interest.
